# Effects of propolis intake on endurance exercise and molecular signaling related to inflammation and oxidative stress

**DOI:** 10.3389/fnut.2025.1539701

**Published:** 2025-02-26

**Authors:** Xiaoying Xu, Bing Hu, Xiaorong Qu

**Affiliations:** ^1^Sports College, Yantai University, Yantai, Shandong, China; ^2^Sports Industry Development Service Department, Yantai Sports Industry Development Service Center, Yantai, Shandong, China; ^3^Training Section, Yantai Shooting and Archery Sports Center, Yantai, Shandong, China

**Keywords:** propolis, inflammatory cytokines, exercise, oxidant status, sport performance

## Abstract

Honey bees extract sticky material from the exudates of different plants which transform afterwards to propolis. Propolis from several global locations has been shown to contain a wide variety of polyphenolic chemicals. Recent studies have revealed that propolis possesses antioxidant, anti-inflammatory, and immunomodulatory abilities. In laboratory animal studies, it has been demonstrated that propolis can enhance the functioning of the antioxidant defense system and decrease the activity of nuclear factor-kappa B. As a result, they can effectively alleviate the damage caused by exercise. One of the main flavonoids found in propolis, quercetin, has been demonstrated to enhance muscle mitochondrial biogenesis and exercise capacity. Propolis may aid athletes in preventing oxidative and inflammatory damage to their muscles during exercise and enhance their athletic performance. The goal of the current review was to evaluate how propolis consumption affected the molecular signaling associated with antioxidant/oxidant state, pro/anti-inflammatory cytokines, and anaerobic/aerobic endurance.

## 1 Introduction

As a result of typical bodily functions and during periods of exercise, individuals innately produce reactive species. These reactive species, such as superoxide, hydrogen peroxide, oxygen singlet, hydroxyl reactive species, and others, are molecules that have unpaired electrons and can react with other substances, causing damage to them. This includes damaging important substances in the body such as DNA, proteins, carbohydrates, and lipids [reviewed in detail in (1, 2)]. These reactive species are known to have detrimental consequences in addition to their beneficial impacts on metabolic processes including hypertrophy and mitochondrial biogenesis. For example, oxidative damage has been linked to increased inflammatory response (3–5), reduced exercise performance (e.g., force generation) reviewed in (6), inflicted muscle damage (5, 7–9), and accelerate fatigue (5, 10–13) if it persists beyond a particular adaption threshold over a lengthy duration. Furthermore, the excessive generation of unstable atoms following vigorous physical exertion can result in permanent harm to the body’s inflammatory response, internal cell structures, and ultimately lead to muscular deterioration (14–17). Finding the optimal equilibrium between the detrimental impact of reactive oxygen species (ROS) on the body and the beneficial effects of adaptive growth can be a challenging endeavor (18, 19). Antioxidants are a collection of substances that have a critical function in safeguarding cells from the negative effects of unstable particles called reactive species (20–22).

The use of nutritional supplements or plant-based diets, which are high in flavonoids, has increased in popularity as a way to stop reactive species damage. There are various subclasses of flavonoids, including isoflavones, flavonols, flavones, and anthocyanidins (23–27). Antioxidant-rich medicinal plants are important for their positive effects and are used as a substitute for traditional therapy for treating oxidative stress-related illnesses. Flavonoids have been found in diverse edibles (such as wine, citrus fruits, and leafy greens) and possess a broad spectrum of physiological effects that could potentially impact disease regulation (28–31).

The honey bees (*Apis mellifera* L.) create a substance called propolis by using the exudates from various plants and buds. Propolis, which is obtained from different locations worldwide, has been shown to contain a multitude of polyphenol compounds (32–34). For many years, propolis has been utilized as a well-liked medicinal substance to support overall wellness and repair wounds and infections (35). Research conducted in multiple global regions has confirmed that propolis possesses appealing characteristics including antioxidant, anti-inflammatory, and immunomodulatory effects (36, 37). Studies on animals have revealed that propolis and its different forms can significantly improve the body’s antioxidant defense mechanism and decrease the function of nuclear factor-kappa B (NF-κB) (38). As a result, this could potentially reduce the likelihood of harm caused by physical activity (39, 40). Based on available data, propolis may aid athletes in preventing oxidative and inflammatory damage to their muscles during exercise and enhance their athletic performance.

## 2 Structure and types of propolis

Honey bees extract sticky material from the exudates of different plants which transform afterwards to propolis. Honeybees utilize it to smooth down the internal walls and fill gaps in honeycombs, strengthening the hive’s structural integrity and keeping outsiders out of the entrance. Propolis in its natural state is composed of a blend of 50% plant resin, 30% beeswax, 10% essential and aromatic oil, and 5% pollen (41, 42). Micro and macro minerals, as well as other chemical substances, make up the remainder. In propolis sourced from various regions, more than eight hundred chemicals have been found (43–45). The components of these substances consist of amino acids, fatty acids, minerals, terpenes, lignans, flavonoids (such as flavones, flavanones, flavonols, dihydroflavonols, and chalcones), and phenolic acids (46, 47). The chemical components makeup of propolis is affected not only by the type of bee and plants from which it is sourced but also by its geographical region (33, 48–50). Propolis is composed mainly of fatty acids (both saturated and unsaturated, making up around 24–26% of the total composition), flavonoids (around 18–20%), and simple sugars (ranged from minimal amounts to ~32%). Small amounts of other substances, such as aromatic acids (5–10%), esters (2–6%), vitamins (2–4%), alcohol and terpenes (2–3.3%), microelements (0.5–2.0%), and other miscellaneous compounds (21–27%). These components, although individually minor, collectively contribute to a significant portion of the total composition, highlighting the complex nature of the propolis. There are around thirty elements known to exist, the most common being Calcium (Ca), Magnesium (Mg), Zinc (Zn), Copper (Cu), Silicon (Si), Iron (Fe), Aluminum (Al). Propolis has been shown to include pro-vitamin A (*β*-carotene), B-group vitamins, including B_1_, B_2_, B_6_, niacin, and folate, and vitamins C, D, and E (51–53). There are also trace quantities of the following enzymes: transhydrogenase, maltase, esterase, *α*-and β-amylase, α- and β-lactamase, and largely from the bee glandular fluid and maybe from pollen (54). According to estimates, the typical total protein concentration of propolis ethanol extract (EEP) is 2.8% (55, 56). Pyroglutamic acid, a derivative of amino acids found in bees, has been discovered in propolis in recent years (57–59). Additionally present are, glucose, rhamnose, fructose, ribose, saccharose, gulose and talose, as well as poly-, di-, and monosaccharides. Poplar tree bud resin, sourced primarily from Populus species, serves as the key element of propolis found in North America, Europe, and parts of Asia outside of the tropics (60, 61). The chemical structures of diverse kinds of propolis obtained from poplar are exceedingly alike. The predominant elements consist of phenolic compounds including aromatic acids, esters, and aglycones flavonoids. The Asteraceae plant, Baccharis dracunculifolia, is the origin of Brazilian green propolis, commonly known as Alecrim. In Brazil, propolis contains unique compounds such as flavonoids, diterpenes, and lignans that are not present in European propolis. Additionally, it is known to have derivatives of prenylated p-coumaric acid and o-hydroxy-acetophenone (55, 56).

## 3 Propolis and molecular signaling related to inflammation

**Figure 1:**
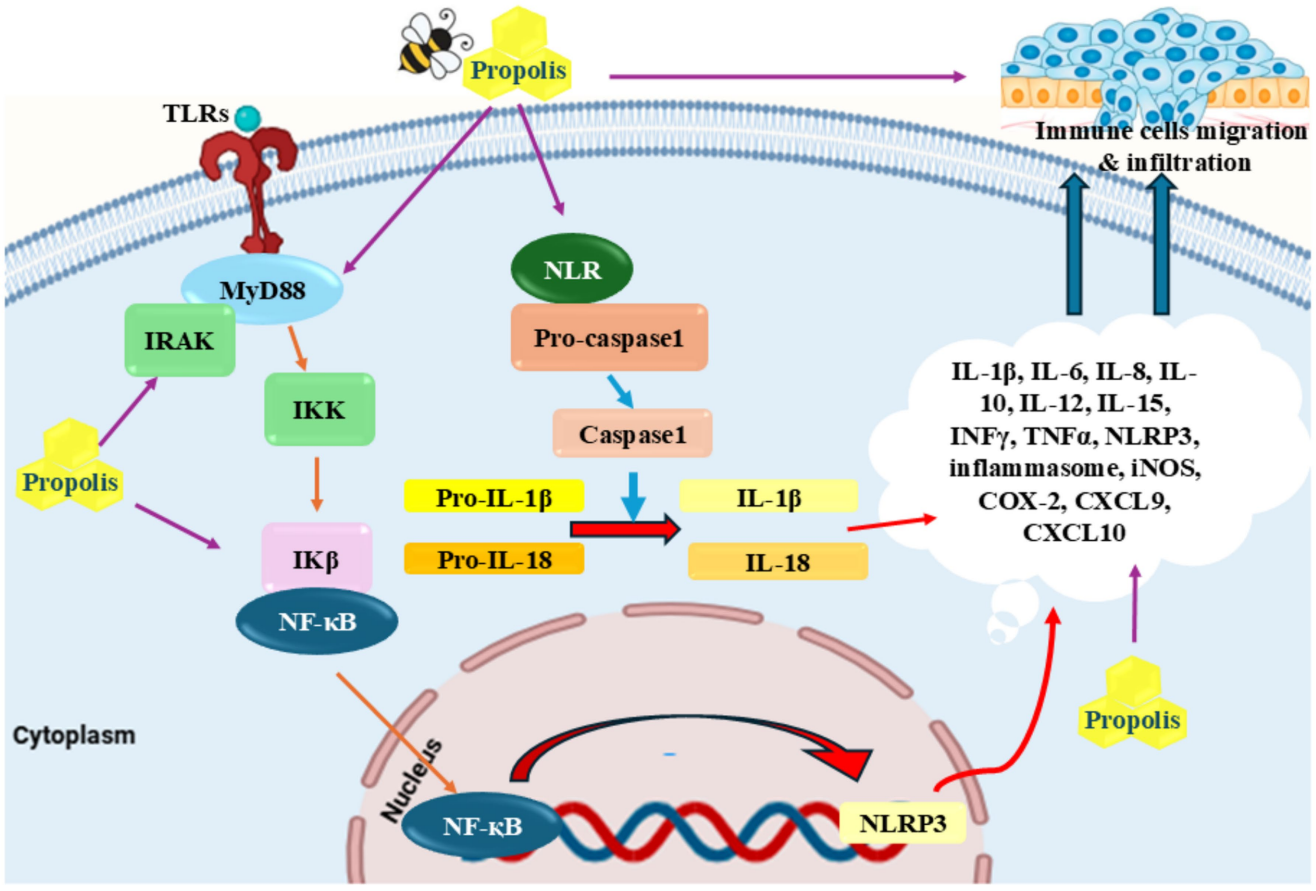
Anti-inflammatory properties of propolis: Propolis inhibited different inflammatory cytokines including TRIF, MyD88, TLR4, and NLRP inflammasomes as well as pro-inflammatory cytokines including TNF-*α*, IL-1β, IL-6, and IFN-*γ*. Reduction of migration of neutrophils and tissue resident macrophages through CXCL9 and CXCL10 is another proposed mechanism for this agent.

The overabundance of reactive oxygen species (ROS), localized disruption of blood flow, and endanger tissue metabolism are some of the many variables that contribute to the complicated process of inflammation. The second stage, known as chronic inflammation, starts if the inflammation lasts for a long period (62). ROS produced via different ways such as smoking, radiation, UV therapy, and unhealthy diet (Figure 1) (63–67). Numerous chronic disorders, such as cancer, diabetes, obesity, and diseases of the heart, lungs, and nervous system are linked to chronic inflammation (68–74). Studies in the fields of clinical and epidemiology have suggested that tumor genesis, development, and progression are all impacted by chronic inflammation (62, 72). Many research on propolis and its constituents have demonstrated that different flavonoids have anti-inflammatory features (75–77). Numerous research has also demonstrated propolis’s anti-inflammatory qualities (78, 79). Numerous studies have demonstrated that propolis effectively acts as a sustainable anti-inflammatory agent, capable of eliminating harmful reactive species in both human and animal cells (78–80). Transcription factors that upregulate pro-inflammatory cytokines can be activated by reactive species. By blocking Interleukin (IL)-8 and IL-6, it has been proven effective in treating food allergies and respiratory conditions. Hyaluronidase activity was shown to be inhibited by propolis extract from different geographic origins *in vitro* (81). Furthermore, the utilization of propolis extracts resulted in a notable reduction in the expression levels of genes associated with the production of pro-inflammatory cytokines, particularly those related to microRNA (miR)-19a-3p, miR-203a-3p, and miR-27a-3p (82) (Figure 1). The various cytokines present were Tumor Necrosis Factor-alpha (TNF-*α*), Interleukin-6 (IL-6), Interleukin-1 beta (IL-1β), and Monocyte Chemoattractant Protein-1 (MCP-1). Also, other molecules such as Intercellular Adhesion Molecule-1 (ICAM-1), and Vascular Cell Adhesion Molecule-1 (VCAM-1) were present. Furthermore, it was found that propolis effectively reduced the expression of Histamine Receptor H1 (H1R) and IL-9 genes and downregulated the protein kinase Cδ (PKCδ) and nuclear factor of activated T-cells (NFAT) signaling pathways in a rhinitis animal model (83). In another experiment involving animals with allergies, it was found that propolis increased the occurrence and amount of polymorphonuclear myeloid-derived suppressor cells (PMN-MDSC), while also reducing both the infiltration of eosinophils and the synthesizes of IL-13 (84). In human clinical trials, Fudhali et al. (85) demonstrated that the use of propolis compress significantly decreased the frequency of phlebitis occurrences caused by intravenous therapy. Susan et al. (86) have expressed that propolis has the ability to potentially function as a form of therapy for pain after undergoing surgery. In addition, Soleimani et al. (87) proved that consuming propolis had a significant impact on reducing IL-6, a pro-inflammatory cytokine, after completing both the Cooper 12-min run test and the running-based anaerobic sprint test.

## 4 Propolis and molecular signaling related to oxidative stress

**Figure 2:**
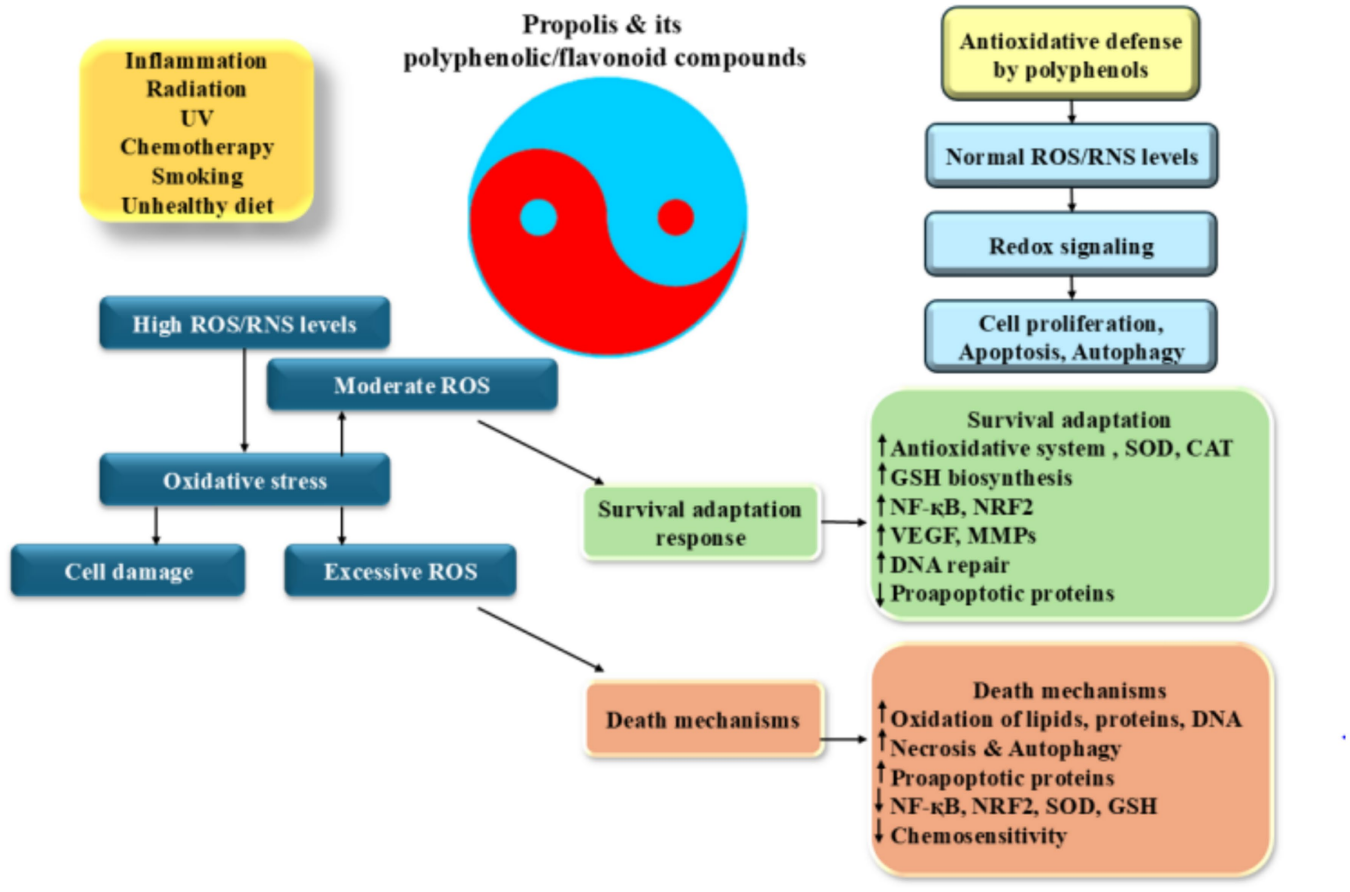
Antioxidative properties of propolis: induction of ROS production which leads to the inflammation increases different factors including HIF-1α, NF-κB, and AP-1. These factors increase pro-inflammatory cytokine production and induction of a wide range of cellular responses. High levels of ROS induces cell death and DNA damage while its moderate levels promote angiogenesis and trigger of DNA repair processes resulted to the cell adaptation and survival. Excess ROS production alters gene expression pattern which induces cancer development and progression.

A wide range of substances, like quercetin, caffeic acid phenethyl ester, caffeic acid, ferulic acid, p-coumaric acid, and chrysin, present in propolis have the ability to decrease the production of molecules that promote inflammation by inhibiting the activity of enzymes that generate reactive oxygen species (ROS), including lipoxygenases, cyclooxygenases, phospholipase A2, and nitric oxidase synthase (44, 88, 89). Moreover, it has been discovered that propolis contains multiple components that effectively hinder the activation of nuclear factor kappa-light-chain-enhancer of activated B cells (NF-κB) and modify the pathways of mitogen-activated protein kinases (MAPKs) and arachidonic acid, preventing them from entering the nucleus. Triggering the NF-κB pathway continuously leads to growth in majority of cancerous cells, whereas inhibition of NF-κB effectively halts the process of cell division (90). The molecular structure of flavonoids and phenolic acids has been demonstrated to positively correlate with antioxidant activity in general when significant polyphenol levels are present (58, 91). As previously demonstrated, flavonoids possess significant capabilities in scavenging harmful reactive species (92). Additionally, the easy oxidation of their phenolic hydroxyl groups serves to enhance their effectiveness as the antioxidant agents. Flavonoids can be identified by certain features, such as an ortho-hydroxyl group on the B-ring, one or more unattached hydroxyl groups on the B-ring, a C2-C3 double bond in the C-ring of the structure, or a free hydroxyl group at either the C-3 or C-4′ position (93, 94). These characteristics are essential for classifying a compound as a flavonoid, which are responsible for their unique properties, make up the framework of the flavonoid structure. The antioxidative action of flavonoids is significantly influenced by their interactions with redox enzymes that play a role in detoxification processes, notably UDP-glucuronosyl transferase, glutathione S-transferase, and NAD (P)H-quinone oxidoreductase. This activity is a crucial aspect of how flavonoids function. These particular proteins play a key role in defending the body from harm caused by oxidative stress (95–98) (Figure 2).

## 5 The outcome of propolis on exercise performance, and molecular signaling related to inflammation, and oxidative stress

### 5.1 Propolis and exercise: clinical trials

Numerous research studies have thoroughly examined the possibility of propolis to reduce problems related to elevated concentration of oxidative stress and inflammation, besides to improve fat metabolism by targeting transcription proteins (99, 100). These effects ultimately contribute to increased physical activity and athletic performance. Soleimani et al.’s research was meticulously conducted to analyze the impact of propolis on inflammation, oxidative stress, and athletic performance in individuals who were not only in good health but also actively engaged in physical activities (87). Thirty-four male military cadets participated in this research investigation and were randomly assign to either receive a 450 mg dose of propolis twice a day for 4 weeks or a placebo made of microcrystalline cellulose. The Cooper 12-min run test and the running-based anaerobic sprint test were employed as methods for evaluating both aerobic and anaerobic capabilities. Shortly after Cooper’s test, blood samples were immediately collected to evaluate markers of both oxidative stress and inflammation. The investigation of fat and fat-free body composition was conducted using bioelectrical impedance. After accounting for initial measurements, the study found no significant differences between the group receiving a 450 mg dose of propolis and the placebo group in the average increases in VO2 max, fatigue index, anaerobic capacity, fat-free mass, and fat mass. However, significant differences were found in the plasma levels of interleukin-6 (IL-6), overall antioxidant capacity, total oxidant status, malondialdehyde, oxidative stress index, and glutathione after adjusting for baseline values and weight fluctuations. Specifically, the propolis group showed a notable contrast compared to the placebo group. Despite no substantial variations in the amounts of interleukin-10 (IL-10) between the two groups, the propolis group exhibited a significantly lower ratio of IL-6 to IL-10 than the placebo group (87).

According to Rashvand et al. (101), taking propolis supplements throughout 28 days considerably impacted the levels of the hormone betatrophin in male endurance athletes’ blood. This hormone is often used as a target in treating dyslipidemia. Four groups were randomly assigned to 44 male athletes: control, physical exercise, placebo, and supplementation. Following the midday and evening meals, both the supplementation and placebo groups were provided with two tablets containing 500 mg each of propolis and cellulose. These tablets were indistinguishable from the original supplement in terms of appearance and color, but did not contain any added taste or scent. Four weeks were spent on the medication regimen. At the start and finish of the four-week course of therapy, the athletes’ weight and blood betatrophin levels were assessed. The weight and betatrophin blood levels of the subjects changed significantly as a result of long-term endurance exercise and propolis supplementation (330.70 ± 35.39 ng/dL and 476.19 ± 59.7 ng/dL for Betatrophin, and 73.75 ± 7.7 kg and 73.45 ± 7.50 kg for weight in pretest and posttest, respectively), whereas similar changes did not occur in the athletes who did not take supplements (300.87 ± 50.59 ng/dL and 299.85 ± 45.44 ng/dL for Betatrophin, and 74.33 ± 1.23 kg and 74.25 ± 1.13 kg for weight, respectively) (101).

Diabetes is characterized by consistently elevated levels of sugar in the blood. However, the combination of proper nutrition and physical activity can effectively reduce the levels of glucose in the bloodstream. Moayedi and colleagues investigated the impact of a combination of propolis supplements and exercise for a period of 8 weeks on the glycemic markers of women with type 2 diabetes mellitus (T2DM). The study involved a total of sixty women with T2DM, who were divided into four groups of fifteen: placebo, exercise and placebo, exercise and propolis, and propolis alone. For 8weeks, group 3 and 4 were administered 500 mg capsules of propolis thrice a day (in the morning, at lunch, and at night) after every meal. Furthermore, Group 2 and 3 participated in three combined exercise sessions (including both aerobic and resistance exercises) per week. The combined training consisted of resistance exercises performed at 60–85% of maximum repetition and aerobic training at 50–70% of maximum heart rate. The study’s findings showed that propolis ingestion, along with exercise, significantly lowered insulin, insulin resistance (IR), fasting blood glucose (FBG), and glycosylated hemoglobin at the same time. Additionally, reducing FBG, insulin, IR, and glycosylated hemoglobin was more significantly impacted by exercise combined with propolis ingestion than by exercise alone (102). The impact of propolis and 8 weeks of aerobic exercise on lipid peroxidation in obese males was studied by Hasanvand et al. (103). The thirty-six participants were chosen using a basic random selection method. Each of the four groups—aerobic exercise, propolis supplement, aerobic exercise + propolis supplement, and control group—was randomly assigned to nine people. Following the initial pretest blood sample collection on the day of the main session, the first group underwent 8 weeks of aerobic exercise. Two 500 mg propolis and one placebo pill, respectively, were given twice a day to each of the two groups. Results indicated that while propolis and aerobic exercise decreased malondialdehyde, triglycerides, and cholesterol in obese individuals, glutathione was unaffected (103).

Another study looks into how young, skilled gymnasts’ performance and a few blood biochemical markers are affected by blends of honeybee products. For 4 weeks, twenty-four juvenile gymnasts, ages six to twelve, were monitored while they went about their regular training regimens. Three groups were formed out of the gymnasts. Group 2 participants were administered a blend of 50 g/day of honey and bee pollen, whereas in the other group, individuals were given a mixture of honey, bee pollen, royal jelly, and propolis. As the control group, group 3 received a 50 g/day dosage of a placebo consisting of wheat starch. Young gymnasts’ biochemical and performance markers were identified at the start and conclusion of the study. Group 2’s grip force and muscle strength metrics increased following therapy. Following supplementation, there was an improvement in the muscle endurance tests conducted in the two treatment groups of honeybee product mixes. Compared to the other groups, group 1’s power test improvement was noticeably greater. Except for the placebo group having lower total protein levels, there were no notable distinctions witnessed between the treatment and placebo groups for any of the studied biochemical markers. Despite receiving supplements containing bee products for a brief duration, the gymnasts demonstrated improved performance in several metrics including muscle endurance, performing pull-ups and seat-ups as well as long jump (104).

It has been reported that dyslipidemia is an imbalance of different lipids, and prior research suggests that propolis may help with this disease. In a single-blind, randomized study, forty-five women with dyslipidemia and T2DM were split into four groups: Patients in the (1) the control group did not use a combination of training and 500 mg capsules of propolis for supplementation; (2) subjects received 500 mg propolis supplement capsules (SUPP); (3) subjects received 500 mg propolis supplement capsules (EXR); (4) subjects received 500 mg propolis supplement capsules (SUPP) in addition to performing combined training. The levels of total antioxidant capacity (TAC), adiponectin, Malondialdehyde (MDA), Secreted Frizzled-Related Protein 5 (SFRP5), and C1q/TNF-Related Protein 12 (CTRP12), IL-6, superoxide dismutase (SOD), and MDA were assessed both before and after the intervention. The results indicated improvement in the levels of MDA, IL-6, TAC, adiponectin, CTRP12, SFRP5, IL-6, and lipid profiles in the EXR + SUPP group (105).

NAFLD is currently recognized as the most widespread form of persistent liver illness, with the potential to be deadly. Researchers conducted a comprehensive investigation to assess the impact of Iranian propolis extract and high-intensity interval training (HIIT) on the levels of serum alanine aminotransferase (ALT) and aspartate aminotransferase (AST) enzymes in patients with non-alcoholic fatty liver disease. Eighteen women and fourteen men with non-alcoholic fatty liver disease (NAFLD) were randomly assigned to one of four groups: high-intensity interval training (HIIT), propolis supplement, propolis supplement combined with HIIT, or control. The individuals in the HIIT group engaged in 8 weeks of exercise that involved one-minute intervals at 80–85% of their maximum heart rate, followed by 2 min at 50–55% of their maximum heart rate. Propolis supplements were administered in the form of 50 mg tablets three times a day, following the main meals. Prior to the intervention, 2 days following the previous training session and supplementation, and before the intervention, body composition, serum AST, and ALT levels were assessed. The ALT and AST enzyme levels were found to have dramatically decreased, according to the results. In the propolis + HIIT groups, there was also a notable drop in enzyme levels. There were no notable alterations observed within either the propolis or control groups for any of the enzymes. The propolis and HIIT group and the HIIT-only group showed a clear distinction from the control group (106). Human serum albumin (HSA) is responsible for scavenging a significant amount of reactive oxygen species (107). This protein is made up of a mix of both reduced human mercapt (HMA) and oxidized nonmercaptalbumin (HNA). Research was conducted on male Japanese fencing (kendo) athletes utilizing a high-performance liquid chromatographic (HPLC) system with an ES-502 N column to examine the redox status of HSA before and after a four-day intensive training camp. Subjects were split into two groups to better understand the propolis supplementation’s antioxidant effect on the HSA redox status throughout camp. Brazil produced the propolis utilized in this trial, which had a daily dose of 787.5 mg. The mean values of [HMA/(HMA + HNA)] (f (HMA)) in the placebo group showed a substantial reduction during camp. Likewise, such ideals had significance for the propolis group as well. However, when looking at the results, it can be seen that the propolis group experienced a significantly smaller decrease in their f (HMA) value compared to the group that received a placebo. These results imply that propolis can be a useful supplement to enhance the redox status of HSA in kendo players during intense, frequent training sessions (108).

Increased blood concentrations of liver enzymes are one of the signs of aging processes, heart disease, cancer, and damages at the cellular level brought on by aerobic and vigorous exercise that raises reactive species. Another research looked at how 4 weeks of aerobic training and propolis supplementation affected endurance athletes’ liver enzyme activity, namely ALT, AST, and SOD. A group of thirty-two male track and field athletes were divided into three randomized groups: one group performed only exercise, another group did exercise along with taking a placebo, and the third group did exercise while taking supplements. The supplements consisted of two 500 mg propolis tablets, taken twice daily. Over the course of 4 weeks, the athletes completed 24 aerobic workout sessions, maintaining a heart rate of 60–65% during each session. The findings indicated a substantial difference in AST, SOD, and ALT levels among the groups. Despite the presence of two separate groups, the placebo and exercise group compared to the exercise-only group did not show any significant discrepancies in terms of AST, SOD, and ALT levels (109). Table 1 summarized the clinical trials conducted in this field.

**Table 1:** The effects of propolis on exercise performance, and molecular signaling related to inflammation, and oxidative stress in human studies.

Participants	Duration	Dosage	Method	Effect	Ref
34 male military cadets	4 weeks	450 mg propolis taken twice daily	To measure both aerobic and anaerobic performance, the Cooper 12-min run test and the running-based anaerobic sprint test were used.	No effect on fatigue index, VO2 max, anaerobic capabilities, fat mass, and fat-free mass, interleukin-10. Reduced of total antioxidant capacity, interleukin-6, total oxidant status, malondialdehyde, oxidative stress index, and glutathione, IL-6/IL-10 ratio.	Soleimani et al. (87)
32 NAFLD patients	8 weeks	50 mg dose of propolis three times a day	NAFLD patients were divided into four groups: propolis supplements, propolis plus HIIT, HIIT, and controls. The participants completed 8 weeks of HIIT, which consisted of one session of 1-min intervals at 80–95% of maximum heart rate and two minutes at 50–55% of reserve heart rate.	No effect on TRPV4 or CYP2E1 protein in propolis group. Reduced TRPV4 and CYP2E1 in the propolis supplement and HIIT groups.	Irandoust et al. (114)
44 male athletes	4 weeks	500 mg tablets of propolis	Four groups were randomly assigned to groups: physical exercise, control, placebo, and supplements.	Reduced weight and betatrophin levels following long-term endurance exercise and propolis supplements.	Rashvand et al. (101)
60 T2DM women	8 weeks	500 mg capsules three times a day	Participants were divided into four groups: (1) placebo, (2) exercise and placebo, (3) exercise and propolis, and (4) propolis. Groups 3 and 4 received propolis in the form of 500 mg capsules three times a day after each meal for eight weeks. Groups 2 and 3 also performed three sessions of combined training (resistance-aerobic) per week. Combined training was resistance training with an intensity of 60–85% of a maximum repetition and aerobic training with an intensity of 50–70% of the maximum heart rate.	Reduced of fasting blood glucose (FPG), insulin, insulin resistance (IR) and glycosylated hemoglobin in propolis groups. Exercise with propolis consumption compared with exercise and propolis had a more significant effect on lowering FBG, insulin, IR, and glycosylated hemoglobin.	Moayedi et al. (102)
38 obese men	8 weeks	500 mg twice daily	Participants were randomly divided into four groups of nine individuals: aerobic exercise, propolis supplement, aerobic exercise + propolis supplement and control group.	Aerobic exercise combined with propolis reduced cholesterol, triglyceride and malondialdehyde but had no effect on glutathione.	Hasanvand and Seif (103)
24 young trained gymnasts	4 weeks	50 g/day with a mixture of honey, bee pollen, royal jelly and propolis	The gymnasts were divided into three groups. Group 1 was administered a mixture of honey and bee pollen at a dose of 50 g/day, whereas group 2 was provided with a mixture of honey, bee pollen, royal jelly and propolis. Group 3 served as the control group and was administered a placebo of wheat starch at a dose of 50 g/day.	No significant difference between treatment and placebo groups in all investigated biochemical parameters except total protein, which was lower in placebo group.	Saritaş et al. (104)
60 women with T2DM and dyslipidemia		500 mg/day	Subjects were divided into four groups: (1) the patients who did not apply the combined training and 500 mg propolis capsules supplement (control group); (2) subjects performed combined training, including aerobic and resistance training (EXR); (3) subjects received the 500 mg propolis supplement capsules (SUPP); (4) Subjects performed combined training along with receiving the 500 mg propolis supplement capsules (EXR + SUPP).	Improved MDA, TAC, IL-6, CTRP12, SFRP5 IL-6, adiponectin, and lipid profile levels in the EXR + SUPP group.	Moayedi et al. (105)
32 NAFLD subjects	8 weeks	50 mg tablet 3 times a day	NAFLD subjects were assigned into four groups of HIIT, propolis supplement, propolis +HIIT and control groups. The subjects participated in 8 weeks of HIIT one bout of 1-min intervals at 80–85% of the maximal heart rate [HRmax], interspersed by 2 min at 50–55% of the maximal heart rate.	Reduced ALT and AST enzymes in the propolis + HIIT groups. In the control and propolis groups, no significant within-group differences were observed in the amounts of any of the enzymes; but there was a significant difference between the propolis + HIIT group with the control group and the HIIT group with the control group.	Gholamhosseini (106)
Male Japanese fencing (“kendo”) athletes	4 days	787.5 mg/day	In order to clarify the antioxidative effect of propolis supplementation on the HSA redox state during camp, subjects were divided into two groups.	Reduced mean values of [HMA/(HMA + HNA)] (f(HMA)) in the placebo and propolis groups. In addition, the change in the f(HMA) value of the propolis group during the training camp was roughly within the normal range of that of healthy male subjects previously reported.	Imai et al. (108)
32 male athletes	4 weeks	500 mg twice a day	Participants were randomly divided into three groups: exercise group, exercise with placebo group, and exercise with supplement group.	Difference between groups in SOD, AST, and ALT levels. No effect between the exercise group and placebo + exercise group in SOD, AST, and ALT levels.	Mahdavi Asiabar et al. (109)

### 5.2 Propolis and exercise: animal models

The effects of propolis alone were examined on dyslipidemia, left ventricular hypertrophy, and atherogenesis in mice with high cholesterol, as well as its connection to swimming (110). The research included four different test groups, all made up of inactive mice (LDLr−/−) that were given a high-fat diet for 75 days and then exposed to aquatic stress for 5 days a week for 5 min each day. The groups were: NAT, who followed a swimming routine for 1 h five times a week, starting on the sixteenth day of the experiment; PRO, who received an oral solution of propolis extract (70 Microliter (uL)/animal/day) starting on the sixteenth day of the experiment; and HL + NAT + PRO, who both followed the swimming routine and received the propolis solution as mentioned above. Blood was collected for serum lipid measurement after 75 days. It was determined what the ratio was between the animal weight (g) and the ventricular weight (mg). Histological slice of the heart and aorta were treated with picrosirius red and hematoxylin/eosin in order to assess any distortions in structure and size. These sections were then subjected to immunohistochemical analysis using anti-CD40L antibodies to investigate the presence of any inflammatory activity. The HL mice showed signs of significant dyslipidemia, atherogenesis, and left ventricular hypertrophy. A downturn in HDL-cholesterol amount was associated with these circumstances, as was the following start of an inflammatory process within the cardiovascular system, demonstrated through a rise in the level of expression CD40L in the left ventricle and aorta. Propolis and swimming, either separately or together, stopped LVH, atherogenesis, and ventricular and arterial inflammation while also raising HDL-cholesterol and reducing CD40L expression (110).

The effects of exercise training and propolis water extract consumption on antioxidant enzyme activity were examined in another study. Because of this, the animals used in the experiment were separated into four distinct groups: CON, CON+Ex, PA, and PA + Ex. During the 6 weeks, every group engaged in exercise through training sessions of 70% VO2max treadmill running for 60 min, 5 days a week. Along with this, the animals were given 50 mg/kg of the propolis water extract per day. The obtained findings indicated that the two exercise-based groups, namely PA + Ex and CON+Ex, exhibited notably decreased levels of blood glucose and insulin compared to the control group. Nevertheless, a comparison of glycogen levels in liver and skeletal muscle tissue between the exercise group and the control group demonstrated considerably elevated values in the former. After examining the antioxidant enzyme levels in the liver and skeletal muscle among the different experimental groups, it became clear that the PA + Ex group displayed notably heightened SOD, GPX, and CAT activities in the gastrocnemius muscle tissue of the animals. Furthermore, the liver tissue’s SOD activity revealed that only the PA + Ex group had a substantial rise, whereas the GDX activity in the PA and CON+Ex groups was considerably greater than in the CON group. The levels of CAT activity observed in the liver tissue showed no differences between the experimental groups. The comparison of MDA levels in the liver tissue, which serves as a measure of tissue damage caused by oxygen reactive species, did not reveal any distinguishable differences among the groups. Regardless, the only noticeable decrease in skeletal muscle tissue was observed in the PA + Ex group, in contrast to the other groups used in the experiment (39).

Athletes frequently utilize anabolic androgenic drugs to enhance their athletic performance. Abuse of these drugs has been linked to reports of kidney injury. A research project was carried out to examine the impact of propolis intake on the functioning of glutathione peroxidase (GPX) in the kidney tissue of male rats who were administered testosterone enanthate and underwent an eight-week course of resistance training. Thirty-two male mice, aged 8 weeks, were used in this investigation. The mice were randomly assigned to four groups after 2 weeks of acclimation and twice-weekly weight assessments: Control (C), sham (RT), resistance training + testosterone (RT + T), and resistance training + testosterone + propolis (RT + T + P). Five days a week of resistance training were combined with 2 days off to complete the regimen. During the initial week, the weights used were equivalent to 60% of the individual’s body weight; thereafter, each week, 20% more weight was added. A dose of 20 mg of testosterone enanthate was administered intramuscularly to rats on steroids. By gavage, the receiving group was given 400 mg of propolis cleaner. Kidney tissue was taken for GPX content following dissection. The exercise + testosterone (110 ng/mL) and exercise + testosterone + propolis groups’ GPX levels (118 ng/mL) have considerably dropped in comparison to the control and sham groups, according to the results (162 and 141 ng/mL, respectively). According to the research, administering testosterone may be an effective method for rising level of oxidative stress markers in the kidney tissue of rats that have undergone resistance training. Propolis has not, in the short term, achieved the expected impact when used to alleviate the adverse effects of steroids (111).

Individuals with diabetes, particularly type 2 diabetes, are at significantly higher risk of cardiovascular disease, with a prevalence 2 to 4 times greater than in those without diabetes. Insulin plays a crucial role in vascular function by modulating vascular tone through two pathways: endothelin-1 and nitric oxide release. Vascular endothelial cells produce endothelin-1, a potent vasoconstrictor that has a contraction effect 10 times stronger than neuropeptide Y, vasopressin, and angiotensin II combined. Angiotensin-converting enzyme activity ultimately leads to the production of angiotensin II, which promotes myocyte hypertrophy, smooth muscle cell proliferation within arterial walls, and the release of platelet-derived growth factor. Each of these processes contributes to the development of vascular diseases. Given the oxidative stress and inflammation associated with diabetes, several interventions have been explored to mitigate these effects. Studies suggest that royal jelly may help alleviate diabetes-related complications by improving metabolic and vascular health.

One potential therapeutic approach to preventing insulin resistance—which raises blood pressure in diabetic patients—is to use royal jelly. Bees acquire an alternate form of sticky material called propolis by gathering it from various tree buds, which is later utilized in covering the exterior of their hive and filling any crevices or spaces in the framework. In research, the indicators of angiotensin-2 and endothelin-1 in the cardiac tissue of ovariectomized rats with diabetes or without evaluation were significantly affected by 56 days of endurance training with royal jelly. In the present experimental research, a total of 40 rats with surgically removed ovaries and diabetes induced by administering 40 mg/kg of streptozotocin were randomly sorted into three groups: The Ovariectomized group (OVXD), the Sham-operated group (Sham), and the Exercise Training (ET) combined with Royal Jelly (RJ) and Propolis treatment (Pr) groups (ET + RJ + Pr), with the remaining five treatment groups being the RJ, Pr, ET, ET + RJ, and ET + Pr groups. So that examine the impact of diabetes and ovariectomies, a control group of six healthy rats was utilized. For 8 weeks, the endurance training groups met for three times a week for 60 min each, with an emphasis on 55–75% VO2 max on the treadmill. The groups who consumed propolis and royal jelly were given peritoneal injections of supplements of 100 mg/kg every day. Results indicated that compared to the OVXD group (4.27 ± 0.34), ET + RJ + Pr group considerably reduced angiotensin-2 (1.57 ± 0.45). The concentrations of angiotensin-2 and endothelin-1 were substantially decreased in the group treated with ET + RJ, in comparison to the OVXD group. Furthermore, the concentrations of angiotensin-2 and endothelin-1 were remarkably reduced in the group given ET + Pr compared to the OVXD group. The amount of endothelin-1 and angiotensin-2 were markedly decreased in the group that received royal jelly, exercise, and propolis compared to the group that only received the medication OVXD. Additionally, the combination of royal jelly and exercise, as well as propolis and exercise, were found to have a stronger impact on reducing the levels of endothelin-1 and angiotensin-2 compared to using royal jelly, endothelin, or propolis alone (112).

The primary focus of researchers has been on leveraging the advantages of physical activity in order to optimize cellular metabolism, as the integration of nutrition and incorporation of natural antioxidants alongside exercise has proved to be highly impactful. Another research looked at how propolis and endurance training protected diabetic ovariectomized rats’ hearts from oxidative and myocardial damage. 36 female Sprague Dawley rats, weights were within the range of 200 to 250 grams and aged between 12 and 16 weeks, were used in this experimental investigation. The group under control, which was healthy, comprised six rats. Thirty ovariectomized rats received an intraperitoneal injection of 40 mg/kg streptozotocin to produce diabetes. The diabetic animals were then split up into five groups of six, which included propolis, sham, endurance training, and endurance training with propolis, as well as diabetic ovariectomized control. Rats in the training groups received five sessions a week of training at a VO2 max of 55–75% for 8 weeks. Additionally, propolis was injected subcutaneously at a rate of 100 mg/kg every day. Pro-oxidant-antioxidant balance (PAB) as well as NF-κB and HSP72 gene expression levels were assessed. The study’s findings demonstrated that compared to the control ovariectomized diabetic group (0.4), the propolis, endurance training, and endurance training plus propolis groups had considerably greater levels of HSP72 expression (0.9, 1.2, 1.4, respectively). The ovariectomized diabetic rats showed a considerable drop in NF-kB and malonaldehyde levels (113).

Table 2 represented some of the studies conducted on animals in the field of propolis and exercise.

**Table 2:** The effects of propolis on exercise performance, and molecular signaling related to inflammation, and oxidative stress in animal studies.

Animal model	Duration	Dosage	Method	Effect	Ref
Hypercholesterolemic mice	75 days	70 uL/animal/day	The experiments were performed in LDLr−/− mice, fed with high fat diet for 75 days, and were divided into four experimental groups (n = 10): HL, sedentary, subjected to aquatic stress (5 min per day, 5 times per week); NAT submitted to a swimming protocol (1 h per day, 5 times per week) from the 16th day of the experiment; PRO, sedentary, submitted to aquatic stress and which received oral propolis extract (70 uL/animal/day) from the 16th day of the experiment; HL + NAT + PRO, submitted to swimming and which received propolis as described above.	The HL animals showed severe dyslipidemia, atherogenesis and left ventricular hypertrophy, associated with a decrease in serum HDL-cholesterol levels and subsequent development of cardiovascular inflammatory process. Swimming and propolis alone and\or associated prevented the LVH, atherogenesis and arterial and ventricular inflammation, decreasing the CD40L expression and increasing the HDL-cholesterol plasmatic levels.	Silva et al. (110)
Rat	6 weeks	50 mg/kg/day, 5 times per week	The exercise training (70% VO_2_max treadmill running exercise for 60 min) of 5 times per week for six weeks and the intake (50 mg/kg/day) of the water extract from propolis were performed by separating the experimental animals into CON group, CON+Ex, PA, and PA + Ex.	Reduced blood glucose and insulin levels of the CON+Ex group and PA + Ex group compared with the control group. Increased the activities of SOD, GPX and CAT in PA + Ex group compared with other experimental groups. Increased the SOD activity in the liver tissue in PA + Ex.	Kwon et al. (39)
Male mice	2 weeks	400 mg	32 male mice with an age of 8 weeks were prepared. After two weeks of familiarization and weighing twice a week, the mice were randomly divided into 4 groups: Control (C), sham (RT), resistance training +testosterone (RT + T) and resistance training + testosterone + propolis (RT + T + P) group were divided. The resistance training program was carried out 5 days a week with two rest days a week.	Reduced GPX levels in the exercise + testosterone and exercise + testosterone + propolis groups compared with the control and sham groups.	Mehrabi et al. (111)
Rats with diabetes	8 weeks	100 mg/kg daily supplements	In this experimental study, 40 ovariectomized rats with diabetes (40 mg/kg streptozotocin) were randomly divided into (1) OVXD, (2) Sham, and (2) groups. (3) RJ, (4) Pr, (5) ET, (6) ET + RJ, (7) ET + Pr, and (8) ET + RJ + Pr were divided. Endurance training groups ran for eight weeks, three sessions a week and each session were 60 min with an intensity of 55–75% VO2 max on the treadmill, especially for rats. The royal jelly and propolis consumption groups received 100 mg/kg daily supplements as a peritoneal injection.	RJ and Pr decreased angiotensin-2 significantly less than the OVXD group. In the ET + RJ group, the values of angiotensin-2 and endothelin-1 were significantly lower than in the OVXD group. Also, in the ET + Pr group, the values of angiotensin-2 and endothelin-1 were significantly lower than in the OVXD group. In the ET + RJ + Pr group, the values of angiotensin-2 and endothelin-1 were significantly lower than in the OVXD group. The effect of exercise + royal jelly and also exercise + propolis on the increase of angiotensin-2 and endothelin-1 was more favorable than the effect of ET, RJ, and Pr.	Behrouz et al. (112)
Rats with diabetes	8 weeks	100 mg/kg/day	Diabetic animals were divided into five groups of six including diabetic ovariectomized control, sham, propolis, endurance training, and endurance training + propolis. Rats in the training groups trained for eight weeks, five sessions per week, with 55–75% VO2 max. Moreover, propolis was administered 100 mg/kg/day by peritoneal injection.	HSP72 expression was significantly higher in the propolis, endurance training, and endurance training + propolis groups. Reduced NF-kB and malonaldehyde in the ovariectomized diabetic rats.	Dolatabadi et al. (113)
Ovariectomized rats with diabetes	8 weeks	100 mg/kg/day	40 Ovariectomized rats with diabetes (40 mg/kg streptozotocin) were randomly divided into (1) OVXD, (2) Sham, and (2) groups. (3) RJ, (4) Pr, (5) ET, (6) ET + RJ, (7) ET + Pr, and (8) ET + RJ + Pr were divided. Endurance training groups ran for eight weeks, three sessions a week and each session were 60 min with an intensity of 55–75% VO2 max on the treadmill, especially for rats.	Reduced RJ and Pr angiotensin-2 significantly less than the OVXD group. Reduced angiotensin-2 and endothelin-1 in the ET + RJ group. In the ET + Pr group, the values of angiotensin-2 and endothelin-1 were significantly lower than in the OVXD group. In the ET + RJ + Pr group, the values of angiotensin-2 and endothelin-1 were significantly lower than in the OVXD group.	Education and Mahalat (115)

Taken all together, these researches data highlight the potential benefits of propolis in addressing oxidative stress, inflammation, and improving athletic performance. However, there are several limitations to consider. The variability in study designs, participant profiles, and intervention protocols creates inconsistencies that challenge direct comparisons across studies. The dosages of propolis, ranging from 50 mg to 100 mg per day, and delivery methods (oral capsules, subcutaneous injections) lack standardization, making it difficult to determine an optimal dosage for consistent effects. Additionally, many studies focus on specific subpopulations (e.g., diabetic individuals, athletes, or animal models), limiting the generalizability of the findings. While animal studies provide mechanistic insights, their translation to human outcomes remains uncertain. Furthermore, most clinical trials did not calculate the required minimum sample size or further trials such as phase II or phase II have not conducted on this topic, which reduces the statistical power to detect significant differences and raises concerns about reproducibility.

The use of propolis in combination with exercise shows promising results, but the mechanisms underlying its effects are not fully elucidated. The potential interactions between propolis components and pathways like NF-κB and antioxidant enzymes are complex and warrant further exploration. A lack of long-term studies also hinders understanding of chronic supplementation outcomes and safety.

## 6 Conclusion

Propolis supplementation shows promise for enhancing athletic performance by modulating oxidative stress and inflammation pathways. Key mechanisms include inhibition of NF-κB signaling, upregulation of antioxidant enzymes, and regulation of inflammatory cytokines like IL-6 and IL-10. These effects may improve recovery, endurance, and overall performance. To achieve beneficial outcomes, the appropriate dosage and delivery method must be standardized. Based on current studies, doses between 50 and 500 mg daily appear effective, depending on individual needs and activity levels. Future research should prioritize large-scale clinical trials, investigate synergistic effects with exercise, and evaluate the long-term safety and efficacy of propolis supplementation.
